# Combined Anterolateral Ligament Reconstruction Results in Better Knee Stability and More Satisfactory Subjective Outcomes in Non-Athlete Patients Undergoing Revision Anterior Cruciate Ligament Reconstruction

**DOI:** 10.3390/jcm13144087

**Published:** 2024-07-12

**Authors:** Se-Han Jung, Ji-Soo Park, Min Jung, Kwangho Chung, Tae-Ho Ha, Chong Hyuk Choi, Sung-Hwan Kim

**Affiliations:** 1 Arthroscopy and Joint Research Institute, Yonsei University College of Medicine, Seoul 03722, Republic of Korea; drshjung@naver.com (S.-H.J.); colum397@gmail.com (J.-S.P.); jmin1103@yuhs.ac (M.J.); khchung85@yuhs.ac (K.C.); abc0117@yuhs.ac (T.-H.H.); choi8422@yuhs.ac (C.H.C.); 2 Department of Orthopedic Surgery, Gangnam Severance Hospital, Yonsei University College of Medicine, Seoul 06273, Republic of Korea; 3 Department of Orthopedic Surgery, Severance Hospital, Yonsei University College of Medicine, Seoul 03722, Republic of Korea; 4 Department of Orthopedic Surgery, Yongin Severance Hospital, Yonsei University College of Medicine, Yongin 16995, Republic of Korea

**Keywords:** knee ligament, anterior cruciate ligament reconstruction, revision anterior cruciate ligament reconstruction, anterolateral ligament reconstruction, rotational instability

## Abstract

**Background**: Consensus has not yet been reached regarding combined anterior cruciate ligament reconstruction (ALLR) with revisional anterior cruciate ligament reconstruction (RACLR). We aimed to compare the clinical outcomes between patients who underwent isolated RACLR and those who underwent RACLR combined with ALLR. **Methods**: Between June 2010 and June 2021, 49 patients who underwent RACLR were retrospectively reviewed over a 24-month follow-up. Patients were categorized into the isolated RACLR (n = 37, group 1) or combined ALLR group (n = 12, group 2). Clinical outcomes were evaluated with several patient-reported outcome measures (PROMs) and minimal clinically important differences (MCIDs) for each PROM. The side-to-side difference (SSD) of the anterior instability was measured. The pivot-shift test was performed. **Results**: Baseline characteristics showed no differences between the groups. PROMs showed no significant differences between the groups at the 2-year follow-up. Group 2 was superior to group 1 in the MCID achievement rate for Lysholm knee and International Knee Documentation Committee (IKDC) subjective scores at 24 months postoperatively. At the final follow-up, the proportion of IKDC grade A in SSD for anterior laxity was higher in group 2 than in group 1 (58.3% versus [vs.] 18.3%, *p* = 0.009), and the proportion of pivot-shift grade 0 was also higher in group 2 (66.7% vs. 27.0%, *p* = 0.013). The “near return to activity” rate was also higher in group 2 than in group 1 (83.3% vs. 45.9%, *p* = 0.043). **Conclusions**: Combining ALLR with RACLR in non-athletes results in a higher proportion of patients with less mechanical graft failure and satisfactory clinical outcomes.

## 1. Introduction

Anterior cruciate ligament (ACL) reconstruction (ACLR) failure is multifactorial and challenging to attribute to a single cause in clinical practice, with revision rates ranging from 3% to 7% in multicenter studies [1,2]. Various causes for failure following ACLR have been identified, including trauma, technical errors, and graft failure to integrate biologically [3,4,5,6,7]. Consequently, up to 10–20% of patients exhibit residual instability after ACLR, which can lead to failure and necessitate revision ACLR (RACLR) [8,9]. Notably, when RACLR is performed, the rates of residual instability and re-rupture are even higher than those with primary ACLR [10,11,12].

The anterolateral ligament (ALL) has received renewed attention as a secondary stabilizer against the tibia’s anterior translation and internal rotation, as supported by several biomechanical studies [13,14,15]. Currently, ALL reconstruction (ALLR) is widely performed and combined with ACLR, showing favorable long-term results, including a five-fold reduction in re-rupture rates after ACLR [16]. However, indications for ALLR remain controversial, with RACLR being one commonly mentioned indication [17,18]. Recent studies have supported combining ALLR with RACLR, showing reduced graft re-rupture rates [19] and improved return to sports rates in mid-term follow-up studies [20,21]. Lee et al. [20] reported that combining ALLR with RACLR significantly reduced rotational laxity and increased return to sports rates compared with RACLR alone. Recent systematic reviews [22,23] also found that combined reconstruction reduces rotational laxity and re-rupture rates and improves clinical outcomes compared with isolated RACLR. However, a closer review of the comparative studies included in these systematic reviews revealed that the included patients are often heterogeneous, and few studies specify patient activities using the Tegner activity scale. Additionally, no studies have evaluated the minimally clinically important differences (MCIDs), an important parameter for determining surgical outcomes in non-athlete patients.

Therefore, we aimed to compare knee laxity and clinical outcomes, including MCID achievement and return to activity rates, between patients who underwent RACLR combined with ALLR and those who underwent isolated RACLR, particularly in non-athlete patients with low Tegner activity scores. We hypothesized that patients who underwent RACLR combined with ALLR would have superior clinical outcomes compared with those who underwent isolated RACLR.

## 2. Materials and Methods

### 2.1. Ethics Statements

This study was approved by the institutional review board of our institution. Owing to the retrospective nature of the study and the minimal risk involved, patient consent was waived by the institutional review board.

### 2.2. Study Design and Patients

Data from 72 consecutive patients who underwent RACLR by a single orthopedic surgeon between June 2010 and June 2021 were retrospectively reviewed. Surgical indications for RACLR were as follows: (1) re-rupture or insufficiency, (2) anteroposterior (AP) instability (Lachman test result ≥ G2 or Telos Lachman side-to-side difference [SSD] ≥ 5 mm or KT-2000 manual max SSD ≥ 5 mm), (3) rotational instability (pivot-shift test result ≥ G2), (4) persistent subjective symptoms related to knee instability with definite AP or rotational instability, and (5) newly developed meniscal tears with definite AP or rotational instability.

The additional inclusion criteria were as follows: (1) at least 24 months of follow-up and (2) patients who underwent all clinical and radiologic assessments (patient-reported outcome measures [PROMs] and stress radiographs) during the follow-up period. The exclusion criteria were as follows: (1) loss of clinical or radiologic data at any point during the follow-up duration, (2) patients with a high pre-injury activity scale score (Tegner activity scale score > 7), (3) patients who underwent significant concomitant cartilage procedures that can affect the clinical outcomes (autologous chondrocyte implantation), (4) patients who underwent concomitant subtotal meniscectomy, (5) patients with multiple ligament injuries (posterior cruciate ligament ruptures or medial collateral ligament injuries, and posterolateral corner injuries), (6) patients with double-bundle RACLR, and (7) patients with two-staged RACLR owing to widened previous tibial/femoral tunnels.

**Figure 1:**
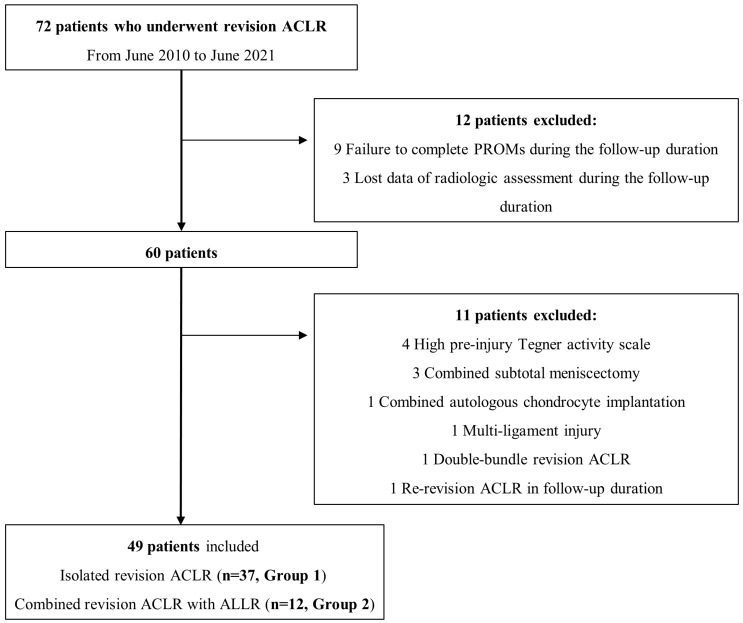
Flowchart of patient inclusion and exclusion in the present study. ACLR, anterior cruciate ligament reconstruction; ALLR, anterolateral ligament reconstruction; PROMs, patient-reported outcome measure scores.

Forty-nine patients who met the stated conditions were included in this study and divided into two groups according to the ALLR procedures: isolated RACLR group (n = 37, group 1) and RACLR combined with ALLR group (n = 12, group 2). Isolated RACLR was performed between June 2010 and January 2019, whereas RACLR combined with ALLR was performed between February 2019 and June 2021 (Figure 1). After a certain time point, ALLR was additionally performed in all patients undergoing RACLR.

### 2.3. Demographics and Baseline Characteristics

Data on patient characteristics, including age, body mass index, sex, and affected side were collected. The profiles of concomitant meniscal tears, chondral lesions, and any concomitant procedures performed were evaluated. Preoperative arthritis was assessed using standard AP knee standing radiographs according to the Kellgren-Lawrence (KL) grading system. The widest diameter of each previous femoral and tibial tunnel was measured using computed tomography (CT) scans, which included coronal and sagittal views [24]. The positions of the previous femoral and tibial tunnel intra-articular orifice were evaluated using a measurement method described in a previous study, using three-dimensional (3D) reconstructed CT scans [25].

### 2.4. Surgical Procedure and Rehabilitation 

*RACLR:* Based on preoperative 3D reconstructed CT scans, the surgical plan involved using the existing tunnels for patients with anatomically positioned previous tunnels, creating independent tunnels for patients with non-anatomically positioned previous tunnels, and implementing the divergent tunnel technique for suboptimal anatomically positioned previous tunnels based on the funnel concept to minimize overlapping while optimizing the angle variations toward the anatomical footprints [26].

For tibialis anterior allografts, 9–10 mm diameter, low-dose gamma-irradiated (12.8–12.9 kGy, cobalt 60) allografts were used. Both sides of the ACL graft were whipstitched using No. 1 Ethibond (Ethicon, Inc., Cincinnati, OH, USA). The graft diameter was measured by folding it in half and passing it through a slot-sizing block. Then, the ACL grafts were pre-tensioned for approximately 20 min at approximately 80 N using a graft preparation board (Graft Master III; Smith & Nephew, Memphis, TN, USA) [27]. For autograft cases, we used quadruple hamstring autografts. The semitendinosus and gracilis tendons were identified near their distal attachments, and any accessory bands along were trimmed. Using an open-loop tendon stripper, the tendons were divided up to the proximal musculotendinous junction. Subsequently, the final semitendinosus-gracilis graft was obtained by detaching the distal attachments from the pes anserine. The harvested hamstring autograft was whipstitched, passed through the loop of the EndoButton CL, and folded in half to form a four-stranded hamstring graft.

The surgical procedure began with diagnostic arthroscopy using the parapatellar high anterolateral portal to assess each knee compartment. Concomitant procedures for meniscal or chondral lesions, if needed, were performed before RACLR. Tibial and femoral tunnels were created on the basis of the previously specified criteria. An additional far anteromedial portal was used for femoral tunnel drilling at 120–130° of knee flexion. The femoral tunnel was drilled to a depth of > 25–30 mm with the same diameter as the ACL graft. Further drilling extended to the far cortex using a 4.5 mm diameter EndoButton CL tunnel reamer (Smith & Nephew, Memphis, TN, USA). Subsequently, the femoral tunnel was further drilled with the ACL tunnel reamer, considering the total length of the femoral tunnel and the EndoButton CL loop diameter.

After creating the femoral and tibial tunnels, the prepared ACL graft was passed through the EndoButton CL loop and folded in half. Using traction on the leading suture of the EndoButton, the EndoButton and ACL graft were guided through the tunnels. Once the ACL graft was completely seated in the femoral socket and the EndoButton CL passed through the femoral cortex, the graft was flipped and confirmed arthroscopically for cortical fixation. Next, a tensioning device (ConMed Linvatec, Largo, FL, USA) was used to apply approximately 70–100 N of tractional force to the graft, which was adjusted on the basis of the graft diameter, and cyclic loading by passive flexion and extension was applied 20 times to optimize tension distribution. Finally, a bioabsorbable interference screw (ConMed Linvatec) was used to fix the graft within the tibial tunnel at approximately 15° of knee flexion with neutral rotation. The outer part of the graft was additionally fixed using a cortical screw and washer.

**Figure 2:**
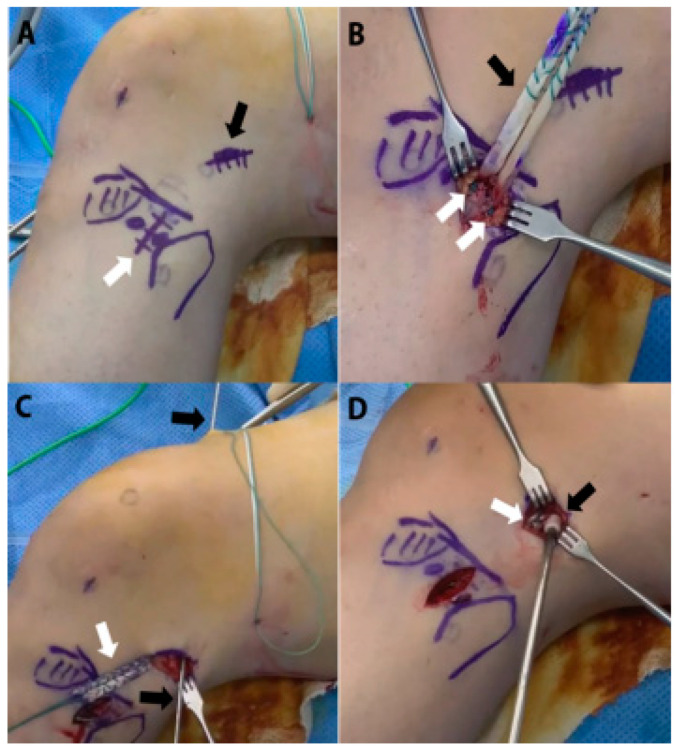
Surgical procedures of ALL reconstruction in combined revision ACL reconstruction. (**A**) Mark the stab incision sites for the femoral lateral epicondyle (black arrow) and the midway area between Gerdy’s tubercle and fibular head at approximately 1 cm distal to the tibial articular surface (white arrow). (**B**) Fixation of the ALL graft using two suture anchors (white arrows) at the tibial attachment site to form a double-arm graft (black arrow). (**C**) After passing the ALL graft (white arrow) under the iliotibial band and positioning it at the femur tunnel site, a 2.4 mm passing pin (black arrows) is drilled into the far cortex of the femoral condyle to form the ALL femoral tunnel. (**D**) After performing ACL graft fixation, the ALL graft (white arrow) that previously passed through the ALL femoral tunnel is fixed with a bioabsorbable interference screw (black arrow) at 15° of knee flexion. ACL anterior cruciate ligament; ALL, anterolateral ligament.

ALLR: Tibialis anterior allografts were used for ALLR. The allograft was trimmed to allow a folded in half, double-stranded graft to pass through the 6 mm diameter tunnels. Both ends of the ALL graft were whipstitched using No. 2 Ethibond, and the graft diameter was measured using a slot-sizing block. ALLR was performed before ACL graft fixation when combined with RACLR. For anatomical ALLR, three key landmarks, the lateral femoral epicondyle (LFE), fibula head, and Gerdy’s tubercle, were marked with the knee flexed at 90° (Figure 2A). A 20 mm incision was made between Gerdy’s tubercle and the fibular head, approximately 10 mm below the joint line, to approach the tibial attachment site of the ALL graft. Two Y-knot suture anchors (ConMed Linvatec) were inserted midway between Gerdy’s tubercle and the fibular head. The mid-point of the prepared ALL graft was positioned at the center of the suture anchor insertion sites and tied to the suture anchor at each point, forming a double-arm configuration on the tibial side (Figure 2B). Next, a 20 mm incision was made at the LFE, followed by longitudinal division of the visible iliotibial band (ITB). A guide pin was inserted at slightly proximal and posterior to the LFE and drilled.

In accordance with reported literature [28], the guide pin was inserted slightly proximal and posterior to the LFE and drilled proximally and anteriorly relative to the trans-epicondylar axis for far-cortex drilling to minimize collision with the pre-existing ACL femoral tunnel. The ALL graft fixed to the suture anchors of the tibia was pulled out under the ITB and positioned at the femur guide pin site. Additionally, a 6 mm diameter cannulated reamer with a 25 mm depth was used to create the femoral socket, followed by drilling through the far cortex with a 2.4 mm Trocar-Tip Passing Pin (Smith & Nephew) (Figure 2C). The ALL graft was passed through the passing pin to the femoral tunnel. Tension was applied to the passing strands exiting the ALL femoral tunnel while assessing the anisometry of the ALL graft (tighter in extension, lax in flexion), and cyclic tensioning was performed on the ALL graft. Finally, a bioabsorbable interference screw (ConMed Linvatec) was inserted to fix the ALL graft at approximately 15° of knee flexion and neutral rotation (Figure 2D).

### 2.5. Clinical Assessment 

Notably, all patients’ clinical and objective stability outcomes were evaluated preoperatively and at 6, 12, and 24 months postoperatively. Clinical outcomes were assessed using the Visual Analog Scale score, Lysholm knee score, International Knee Document Committee (IKDC) subjective scores, and Tegner activity scale score. The Tegner activity scale was used to assess “return to activity,” as in a previous study [29]. We classified the Tegner-related “return to activity” into (1) “return to activity”: Tegner activity scale score at the 24-month follow-up secured the return to pre-injury activity level and (2) “near return to activity”: Tegner activity scale score at the 24-month follow-up returned to pre-injury activity level or reached one level below the pre-injury activity level [29]. Clinically, MCID scores can be used to set treatment thresholds using outcome measures and refer to the change in outcome score resulting in the smallest but most noticeable clinical improvement after surgery [30,31]. We attempted to use MCID thresholds derived from a reliable > 5-year follow-up ACLR-specific study for possible PROMs in our study (Lysholm knee score, 10.6; IKDC score, 9.5) [32]. The objective stability knee joint test assessed anterior instability using the KT-2000 arthrometer (MED Metric, SanDiego, CA, USA) measurements with maximal manual stress. Stress radiography using a Telos device (Metax, Hungen, Germany) at 250 N was used to evaluate SSD; both tests were performed at 30° of knee flexion. We also categorized SSD based on IKDC knee examination form. SSD evaluated using the KT-2000 arthrometer is classified as IKDC A (–1 to 2 mm), IKDC B (3–5 mm), IKDC C (6–10 mm), and IKDC D (> 10 mm), whereas that evaluated using Telos stress radiography is classified as IKDC A (0–2 mm), IKDC B (3–5 mm), IKDC C (6–10 mm), and IKDC D (> 10 mm) [33]. A pivot-shift test was performed, and the result was classified as grade 0 (normal), grade I (glide), grade II (clunk), or grade III (locking) on the basis of the IKDC form to evaluate the rotational instability [34]. All manual examinations were performed by the senior author.

### 2.6. Statistical Analysis

All statistical analyses were performed with SPSS version 26.0 (IBM Corp., Armonk, NY, USA). Continuous variables are reported as mean ± standard deviation, and categorical variables are presented as numbers and percentages unless otherwise specified. In the comparative analysis between the groups, continuous variables were compared using the independent *t*-test or Mann–Whitney U test, according to the results of the normality test (Shapiro–Wilk test). Categorical variables of the two groups were compared using the Pearson chi-squared or Fisher exact tests. Statistical significance was set at *p* < 0.05. Statistical power analysis was performed using G-power (version 3.1, Heinrich Hein University, Dusseldorf, Germany). 

## 3. Results

### 3.1. Patient Demographics and Predisposing Variables

The baseline demographic data showed no significant differences between the groups. Moreover, there were no significant differences between the two groups regarding the diameter and position of the ACL tunnel for primary reconstruction (Table 1). Additionally, the presence, location, and concomitant procedure for the meniscal and cartilage lesions did not exhibit significant differences between the groups, except for the higher rate of both meniscus tears in group 2 compared to group 1 (*p* = 0.029). There was also no difference in the ACL graft selection used in RACLR (Table 2).

### 3.2. Clinical Outcomes 

**Figure 3:**
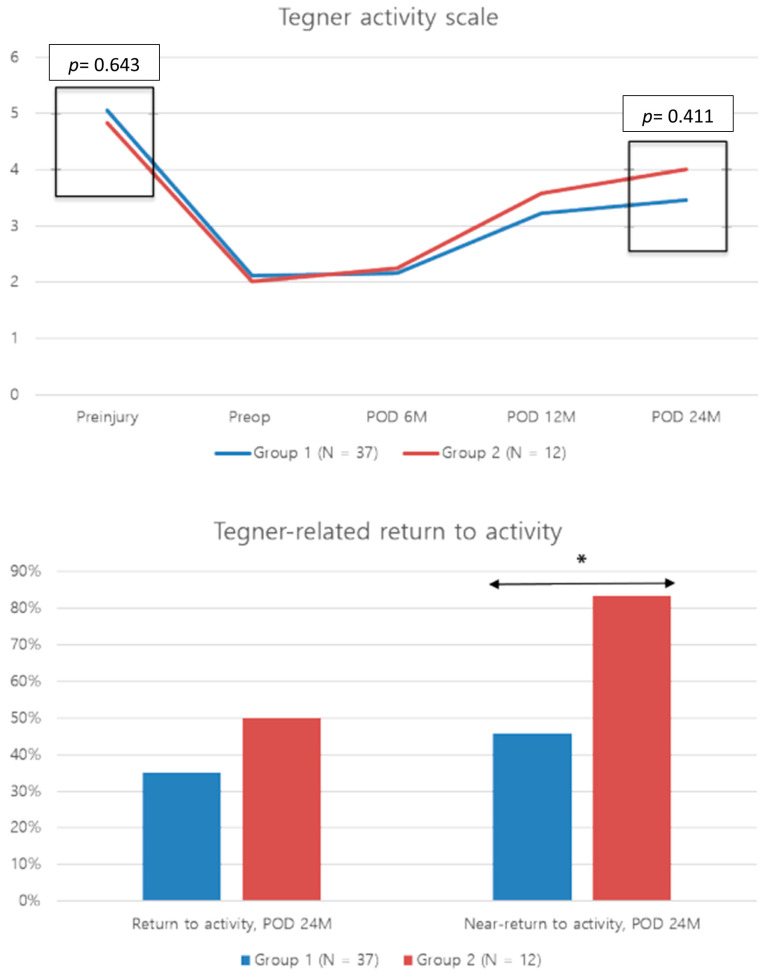
Comparison of pre-injury, preoperative to 24 month postoperative Tegner activity scale score, and Tegner-related “return to activity” rate between the groups. * The significant difference in the Tegner-related return to activity rate at 24 months postoperatively between the groups (*p* < 0.05). Group 1, isolated revision anterior cruciate ligament reconstruction group; group 2, combined revision anterior cruciate ligament reconstruction with anterolateral ligament reconstruction group; POD, postoperative; Preop, preoperative.

Notably, all clinical outcomes in both groups significantly improved 24 months postoperatively compared with preoperative values (*p* < 0.001). Preoperative subjective clinical outcomes were similar between the groups, with no difference observed at any postoperative time point (Table 3). There was no difference in the pre-injury, preoperative, and postoperative 24 month Tegner activity scale scores between the groups. However, the rate of “near return to activity” was significantly higher in group 2 (83.3%) than in group 1 (45.9%) (*p* = 0.043) (Figure 3).

### 3.3. MCID Achievement Rate

**Figure 4:**
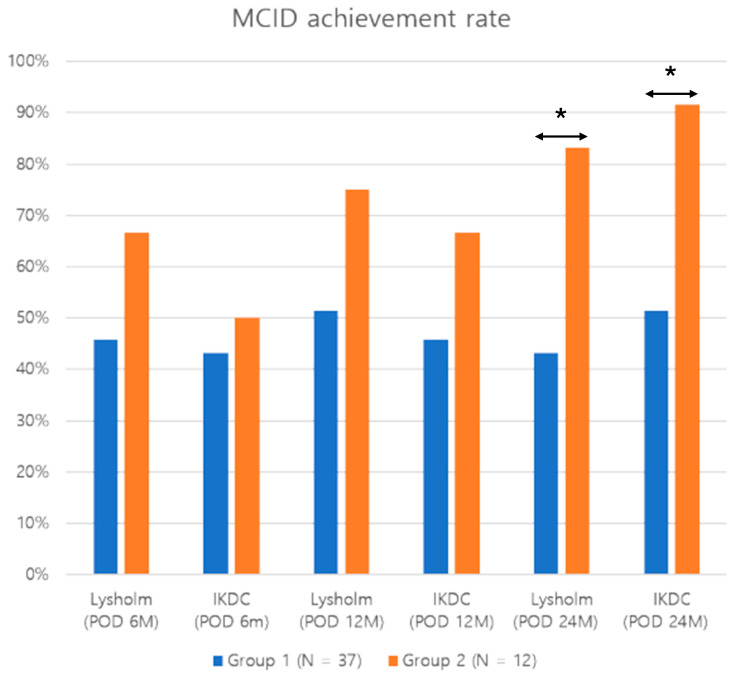
MCID achievement rate of Lysholm and IKDC subjective scores between the groups postoperatively. * Significant differences between the groups at 24 months postoperatively were demonstrated for the Lysholm and IKDC subjective scores (*p* < 0.05). Group 1, isolated revision anterior cruciate ligament reconstruction group; group 2, combined with anterolateral ligament reconstruction group; MCID, minimum clinically important difference; IKDC, International Knee Documentation Committee Subjective Knee Form; Lysholm, Lysholm knee score.

The two groups showed no significant differences in MCID achievement rates for Lysholm knee and IKDC subjective scores at 6 and 12 months postoperatively. However, at 24 months postoperatively, group 2 showed a significantly higher proportion of patients achieving the MCID for the Lysholm knee (83.3% vs. 43.2%, *p* = 0.021) and IKDC subjective scores than group 1 (91.7% versus [vs.] 51.4%, *p* = 0.017) (Figure 4). Statistical power for the significant results regarding clinical outcomes ranged from 0.91 to 0.99.

### 3.4. Objective Ligament Stability

The mean SSD in anterior instability tests did not show a significant difference between the groups preoperatively and at all postoperative time points, despite an overall lower SSD in group 2 than in group 1 (Table 4). The proportions of patients classified into each IKDC grade differed significantly in SSD on Lachman stress radiographs between the groups (Table 5). Additionally, the proportions of IKDC grade A on Lachman stress radiographs and the pivot-shift test were significantly higher in group 2 than in group 1 at 24 months postoperatively (Table 5). Statistical power for the significant results regarding ligament stability ranged from 0.80 to 0.99.

## 4. Discussion

The principal findings of this study are as follows. Combining ALLR with RACLR in non-athletes with low pre-injury activity levels provided additional clinical benefits at the 24-month follow-up. These benefits included a greater MCID achievement rate in several PROMs (Lysholm knee and IKDC subjective scores), a higher “near return to activity rate”, and less residual laxity, with a higher proportion of patients achieving IKDC grade A according to the Lachman Telos stress radiographs and pivot-shift test.

To the best of our knowledge, few studies have analyzed MCID achievements after combined RACLR with ALLR. In this study, PROMs did not differ between the groups at 24 months postoperatively. However, further analysis using MCIDs revealed significant differences in clinical outcomes. Using the MCID values from a previous study [32], we found a significantly better MCID achievement rate in the combined group than in the RACLR-only group. This analysis reduced the ceiling effect of patients with RACLR who had higher preoperative PROMs.

Several studies have compared return to sports rates after primary ACLR and R-ACLR, which reported significantly lower rates in the R-ACLR group than in the primary ACLR group [35,36]. Mohsen et al. [37] reported that over a mean 49-month follow-up of 62 patients, the return to sports rate was 14.5% lower in the revision group than in the primary group. The mean time to return to sports was 35.3 weeks in the revision group compared with 29.2 weeks in the primary group, and the rate of return to the same level of activity before injury was 40.3% in the revision group compared with 54.8% in the primary group. Lee et al. [20] performed a comparative analysis of 87 patients with a mean follow-up of 40 months and reported a significantly higher return to the same level of sports activity in the RACLR combined with the ALLR group (57.1%) than in the isolated RACLR group (25.6%). They also reported that the Tegner score at the last follow-up was significantly better in the RACLR combined with the ALLR group than in the isolated RACLR group (7.0 ± 0.8 vs. 6.3 ± 0.7; *p* < 0.001). However, in this study, the mean pre-injury Tegner activity scale scores of the included patients were even lower than the postoperative Tegner activity scale scores of the patients in the study by Lee et al. [20]. In non-athletic patients, the intensity of activities is often intentionally reduced after surgery to prevent re-injury. Several meta-analyses of patients undergoing RACLR have reported return to sport rates to be as high as 75.0–85.3%, but return to sport rates at pre-injury levels have been reported to be as low as 53.4–57.0% [36,38]. This suggests that it is useful to analyze individual patients’ “near return to activity” when evaluating the outcomes of RACLR in patients who are not professional athletes. In this study, the combined ALLR group demonstrated a significantly higher “near return to activity” rate than the isolated RACLR group, which is a meaningful finding in the non-athlete patient cohort.

Studies on the effectiveness of combined ALLR with RACLR are a topic of continued interest, as graft rupture rates are approximately 14% after isolated RACLR, with persistent rotational instability in approximately 10.0–25.0% of patients [8,39]. Several comparative studies have demonstrated a significant effect of additional ALLR on improving pivot-shift and clinical outcomes [40,41]. Notably, several biomechanical studies have demonstrated the ability of the ALL to resist tibial internal rotation but a reduced ability to resist anterior translation [42,43]. However, recent cadaveric knee studies have shown that ALL injuries can also cause additional effects on anterior translation [14,44]. Helito et al. [40], in their clinical study, found that the RACLR combined with the ALLR group was significantly superior to the isolated RACLR group for mean SSD on KT-1000 arthrometer (1.14–1.6 mm vs. 1.5–2.1 mm, *p* = 0.048) and residual pivot-shift rate (9.8% vs. 35.3%, *p* = 0.011) at the final follow-up. Hamido et al. [45] also reported that at a mid-term follow-up of 5 years in 109 athletes with pivot-shift grade ≥ 2, the rate of residual anterior laxity < 3 mm on KT-1000 arthrometer and pivot-shift grade 0 in the additional ALLR group was 96.0%, and the rate of reaching IKDC grade A was also significantly higher than that in the isolated RACLR group (96.0% vs. 84.6%, *p* < 0.001). These findings align with our study’s results.

In our study, combining ALLR with RACLR did not yield a significant difference in parameters related to anterior laxity (SSD by KT-2000 arthrometer or Lachman Telos stress radiographs). However, a slightly different trend was observed between the groups regarding mean values. Between the 1- and 2-year follow-up periods, anterior laxity remained stable in the combined ALLR group, whereas it increased in the isolated RACLR group. Current literature does not provide a clear explanation for this. Potential causes for the increase in residual laxity include the resolution of knee stiffness and the initial strong graft fixation leading to increased laxity. Additionally, in patients who showed chronic anterior tibial translation, it is thought that the surrounding soft tissue of the knee joint exhibits a trend toward anterior translation of the tibia. In this context, the maintenance of laxity in the combined ALLR group may be attributed to the role of the ALL as a secondary stabilizer [46]. Despite no significant differences in SSDs for anterior laxity, the proportion of patients exhibiting near-normal knee stability, classified as IKDC grade A, was significantly higher in the combined group compared to the isolated group. A similar pattern was observed in the comparison of PROMs between the groups. While PROMs did not show significant differences, the MCID achievement rates were significantly higher in the combined group than in the RACLR-only group. Overall, combining ALLR with RACLR seems to result in a higher proportion of patients with less residual instability and more satisfactory clinical outcomes.

This study has a few limitations. First, it was a retrospective study with a small sample size, introducing the risk of bias. The limited sample size may have reduced the statistical power to detect significant differences between the two groups. However, this study’s results showed clearly significant differences in specific outcomes, countering these concerns. The statistical power for the significant results from this study ranged from 0.80 to 0.99, showing sufficient statistical power. Due to the study’s retrospective nature, there were limitations in obtaining information on primary ACLR surgery or the primary injury, which could potentially influence the outcomes. Such information was not included in the comparative analysis and considered in the inclusion and exclusion criteria. Second, the two groups significantly differed in the incidence of both meniscus tears (*p* = 0.029). Despite this, knee stability outcomes in group 2, which had more instances of both meniscus tears, were better at the final follow-up, making the overall interpretation of the analysis unproblematic. Third, the study’s follow-up period was limited to 24 months, so longer follow-up studies are needed to generalize the results. Lastly, combining ALLR with RACLR was performed more recently than isolated RACLR. This temporal difference could have contributed to the outcome disparity between the two groups, potentially influenced by the surgeon’s improved skills over time. However, this bias may have been mitigated since a single senior orthopedic surgeon performed all surgeries.

## 5. Conclusions

Combining ALLR with RACLR in non-athletes results in a high proportion of patients with less residual instability and satisfactory clinical outcomes, including a higher return to activity rate at the 24-month follow-up.

## Figures and Tables

**Table 1:** Patients’ demographics and predisposing variables.

Variable	Group 1 (n = 37)	Group 2 (n = 12)	*p*-Value
Age, y	29.1 ± 11.1	30.9 ± 12.1	0.633
BMI, kg/m^2^	26.0 ± 4.1	27.4 ± 3.1	0.300
Sex			0.665
Male	31 (83.8)	11 (91.7)	
Female	6 (16.2)	1 (8.3)	
Affected side			0.296
Rt	26 (70.3)	6 (50.0)	
Lt	11 (29.7)	6 (50.0)	
Kellgren-Lawrence grade			0.160
0	13 (35.1)	1 (8.3)	
1	18 (48.7)	7 (58.3)	
2	6 (16.2)	4 (33.3)	
Radiographic parameter ^a^			
Femoral tunnel position			
Depth, %	35.2 ± 9.6	34.3 ± 4.8	0.670
Height, %	25.2 ± 10.5	23.9 ± 15.9	0.804
Tibial tunnel position			
Depth, %	36.5 ± 6.6	38.9 ± 10.6	0.470
Width, %	44.9 ± 5.7	43.6 ± 9.0	0.563
Femoral tunnel diameter, mm ^b^	10.8 ± 2.4	11.5 ± 3.0	0.410
Tibial tunnel diameter, mm ^b^	11.4 ± 2.6	12.7 ± 2.2	0.127

Note. The values are presented as mean ± standard deviation or number of patients (percentage). ^a^ Radiologic parameters measured on computed tomography images taken before surgery. ^b^ Each tunnel measured by the widest tunnel diameter in coronal and sagittal views. BMI, body mass index; Rt, right; Lt, left.

**Table 2:** Concurrent meniscal and chondral lesions and graft type for the RACLR and RACLR with ALLR group.

Variable	Group 1 (n = 37)	Group 2 (n = 12)	*p*-Value
Meniscus tear location			
Medial meniscus	18 (48.6)	8 (66.7)	0.144
Lateral meniscus	12 (32.4)	6 (50.0)	0.486
Both meniscus	4 (10.8)	5 (41.7)	0.029
Medial meniscus procedure ^a^			0.478
None	19 (51.4)	4 (33.3)	
Partial meniscectomy	11 (29.7)	4 (33.3)	
Repair	7 (18.9)	4 (33.3)	
Lateral meniscus procedure ^a^			0.286
None	27 (73.0)	6 (50.0)	
Partial meniscectomy	6 (16.2)	3 (25.0)	
Repair	4 (10.8)	3 (25.0)	
Cartilage defect location			
Medial femoral condyle	6 (16.2)	3 (25.0)	0.669
Lateral femoral condyle	3 (8.1)	3 (25.0)	0.148
Medial tibial plateau	3 (8.1)	2 (16.7)	0.584
Lateral tibial plateau	2 (5.4)	3 (25.0)	0.087
Trochlea	9 (24.3)	1 (8.3)	0.414
Patella	5 (13.5)	0 (0)	0.315
Kissing lesions	4 (10.8)	3 (25.0)	0.340
Multiple lesions	4 (10.8)	2 (16.7)	0.626
Cartilage procedure ^a^			0.369
None	28 (75.7)	7 (58.3)	
Chondral shaving	3 (8.1)	2 (16.7)	
Microfracture or microdrilling	6 (16.4)	3 (25.0)	
Revision ACLR graft type, n			0.500
Autograft/Allograft/Mixed graft	8/27/2	1/10/1	

Note. The values are presented as the number of patients (percentage). Boldface *p*-values indicate a significant difference between the groups (*p* < 0.05). ^a^ Intra-operative concurrent procedures during revision anterior cruciate ligament reconstruction. ACLR, anterior cruciate ligament reconstruction.

**Table 3:** Comparison of preoperative and postoperative clinical outcomes.

Variable	Group 1 (n = 37)	Group 2 (n = 12)	*p*-Value
Preoperative			
VAS score	35.7 ± 22.3	30.6 ± 27.3	0.516
Lysholm knee score	65.8 ± 21.9	58.5 ± 23.6	0.329
IKDC subjective score	51.2 ± 13.3	47.4 ± 24.7	0.620
Pre-injury Tegner activity scale score	5.0 ± 1.4	4.8 ± 1.2	
Tegner activity scale score	2.1 ± 1.7	2.0 ± 1.6	0.844
6 months postoperatively			
VAS score	25.2 ± 20.6	20.4 ± 17.6	0.470
Lysholm knee score	73.5 ± 17.9	76.9 ± 113.4	0.552
IKDC subjective score	55.4 ± 13.9	54.3 ± 14.8	0.815
Tegner activity scale score	2.2 ± 1.2	2.3 ± 1.2	0.826
12 months postoperatively			
VAS score	20.7 ± 15.9	13.9 ± 17.7	0.217
Lysholm knee score	78.4 ± 16.0	85.1 ± 10.4	0.180
IKDC subjective score	61.8 ± 14.1	62.9 ± 11.8	0.816
Tegner activity scale score	3.2 ± 1.9	3.6 ± 1.8	0.561
24 months postoperatively			
VAS score	17.1 ± 16.0	16.7 ± 13.1	0.944
Lysholm knee score	76.3 ± 17.4	85.9 ± 9.9	0.105
IKDC subjective score	66.8 ± 13.9	68.1 ± 13.5	0.792
Tegner activity scale score	3.5 ± 1.9	4.0 ± 2.1	0.411

Note. The values are presented as mean ± standard deviation. ADL, activities of daily living; IKDC, International Knee Documentation Committee; QOL, quality of life; VAS, Visual Analog Scale.

**Table 4:** Side-to-side difference of anterior instability tests from preoperative to postoperative follow-ups.

	Group	Preoperative	6 Months Postoperatively	12 Months Postoperatively	24 Months Postoperatively
KT-2000 arthrometer	Group 1	6.9 ± 2.8	2.1 ± 1.8	1.9 ± 1.7	3.1 ± 2.0
	Group 2	6.7 ± 3.3	2.2 ± 1.1	2.2 ± 3.7	2.1 ± 3.3
		0.817	0.914	0.539	0.213
Lachman Telos stress radiography	Group 1	6.9 ± 4.8	3.0 ± 3.6	3.4 ± 3.0	4.6 ± 3.0
	Group 2	6.8 ± 4.6	1.6 ± 4.4	2.6 ± 4.0	3.3 ± 4.9
		0.960	0.271	0.453	0.266

Note. The values are presented as mean ± standard deviation. Boldface *p*-values indicate a significant difference between the groups (*p* < 0.05).

**Table 5:** Pre- and postoperative ligament stability examination using the IKDC knee examination form.

**Side-to-Side Difference According to the KT-2000 Arthrometer**						
	**Group**	**IKDC A** **(−1–2 mm)**	**IKDC B** **(3–5 mm)**	**IKDC C** **(6–10 mm)**	**IKDC D** **(>10 mm)**	***p*** **-Value**
Preoperatively	Group 1	0 (0)	2 (5.4)	31 (83.8)	4 (10.8)	0.546
	Group 2	0 (0)	1 (8.3)	9 (75.0)	2 (16.7)	
6 months postoperatively	Group 1	19 (51.4)	15 (40.5)	3 (8.1)	0 (0)	0.477
	Group 2	8 (66.7)	4 (33.3)	0 (0)	0 (0)	
12 months postoperatively	Group 1	25 (67.6)	10 (27.0)	2 (5.4)	0 (0)	0.742
	Group 2	9 (75.0)	2 (16.7)	1 (8.3)	0 (0)	
24 months postoperatively	Group 1	11 (29.7)	21 (56.8)	5 (13.5)	0 (0)	0.203
	Group 2	7 (58.3)	4 (33.3)	1 (8.3)	0 (0)	
	*p* value	0.074	0.158	0.634		
**Side-to-Side Difference According to Lachman Telos Stress Radiographs**						
	**Group**	**IKDC A** **(0–2 mm)**	**IKDC B** **(3–5 mm)**	**IKDC C** **(6–10 mm)**	**IKDC D** **(>10 mm)**	***p*** **-Value**
Preoperatively	Group 1	0 (0)	3 (8.1)	27 (73.0)	7 (18.9)	0.573
	Group 2	0 (0)	1 (8.3)	7 (58.3)	4 (33.3)	
6 months postoperatively	Group 1	14 (37.8)	16 (43.2)	7 (18.9)	0 (0)	0.023
	Group 2	9 (75.0)	2 (16.7)	1 (8.3)	0 (0)	
12 months postoperatively	Group 1	13 (35.1)	14 (37.8)	9 (24.3)	1 (2.7)	0.043
	Group 2	9 (75.0)	2 (16.7)	1 (8.3)	0 (0)	
24 months postoperatively	Group 1	7 (18.9)	24 (64.9)	2 (5.4)	4 (10.8)	0.016
	Group 2	7 (58.3)	2 (16.7)	2 (16.7)	1 (8.3)	
	*p*-value	0.009	0.004	0.216	0.805	
**Pivot-Shift Grade**						
	**Group**	**0** **(Normal)**	**I** **(Glide)**	**II** **(Clunk)**	**III** **(Locking)**	***p*** **-Value**
Preoperatively	Group 1	0 (0)	3 (8.1)	23 (62.2)	11 (29.7)	0.400
	Group 2	0 (0)	0 (0)	6 (50.0)	6 (50.0)	
6 months postoperatively	Group 1	22 (59.5)	11 (29.7)	4 (10.8)	1 (9.1)	0.493
	Group 2	8 (66.7)	4 (33.3)	0 (0)	0 (0)	
12 months postoperatively	Group 1	18 (48.6)	12 (32.4)	7 (18.9)	0 (0)	0.265
	Group 2	7 (58.3)	5 (41.7)	0 (0)	0 (0)	
24 months postoperatively	Group 1	10 (27.0)	15 (40.5)	11 (29.7)	1 (2.7)	0.090
	Group 2	8 (66.7)	3 (25.0)	1 (8.3)	0 (0)	
	*p*-value	0.013	0.332	0.134	0.565	

Note. The values are presented as the number of patients (percentage). Boldface *p*-values indicate a significant difference between the groups (*p* < 0.05). IKDC, International Knee Documentation Committee.

## Data Availability

The data presented in this study are available on request from the corresponding author.
